# Headless Screw Fixation Is Associated with Reduced Hardware Removal After Tibial Tubercle Osteotomy: A Retrospective Cohort Study

**DOI:** 10.3390/jcm15010235

**Published:** 2025-12-28

**Authors:** Oguzhan Uslu, Ozkan Kose

**Affiliations:** Department of Orthopedics and Traumatology, Antalya Training and Research Hospital, University of Health Sciences, 07100 Antalya, Türkiye; oguzhannuslu@gmail.com

**Keywords:** tibial tubercle osteotomy, patellofemoral instability, headless compression screw, hardware removal, complication, reoperation

## Abstract

**Background/Objectives**: Symptomatic hardware removal remains the most frequent cause of reoperation after tibial tubercle osteotomy (TTO), with removal rates reported as high as 49%. Headless compression screws have been proposed as a low-profile alternative to conventional screws to reduce hardware-related morbidity, yet no study has directly compared their use with headed screws in TTO for patellofemoral instability. This study aimed to compare complication rates and the frequency of hardware removal between headless and headed screw fixation in TTO. **Methods:** A retrospective review was conducted on 84 patients (94 knees) who underwent TTO between 2014 and 2024. Patients were divided into two groups based on the type of fixation used: headless screws (56 knees) and headed screws (38 knees). Demographic characteristics, perioperative variables, functional outcomes (Kujala, Lysholm, and Tegner scores), complications, and reoperation rates were compared with a minimum one-year follow-up. **Results:** No significant differences were found between the groups in terms of baseline demographic and clinical characteristics. Symptomatic implant removal occurred in 13.2% of the headed screw group and in none of the patients in the headless screw group (*p* = 0.001). Reoperation for any reason was significantly lower in the headless group (3.6% vs. 26.3%, *p* = 0.002). Functional outcomes were similar between groups. Post-hoc power analysis confirmed sufficient statistical power (98.8%) to detect differences in implant removal rates. **Conclusion:** Headless screw fixation in TTO was associated with significantly lower rates of hardware-related reoperations and painful implant removal, while achieving functional outcomes similar to those with headed screws. Headless screws may represent a preferable fixation method for reducing implant-related complications in TTO.

## 1. Introduction

Acute patellar dislocations are common in adolescents and often lead to functional limitations, with recurrence affecting nearly one-third of patients at long-term follow-up [1,2,3]. Key risk factors for acute and recurrent patellar dislocations include young age, open physes, trochlear dysplasia, patella alta, and an increased tibial tuberosity–trochlear groove (TT–TG) distance [2,3,4]. The medial patellofemoral ligament (MPFL), which provides approximately half of the soft-tissue restraint against lateral displacement [5], is ruptured in most first-time dislocations, with a prevalence of up to 94.7% [6].

Non-operative treatment may be considered after a first-time dislocation in the absence of repairable osteochondral injuries; however, recurrent dislocations typically require surgical intervention [7,8]. Surgical management is individualized according to the patient’s anatomical risk factors. The medial patellofemoral ligament reconstruction (MPFLR) is regarded as the cornerstone of surgical management, given the high incidence of MPFL rupture and its pivotal role in medial patellar stability. MPFLR is often supplemented with tibial tubercle osteotomy (TTO) in patients with increased TT–TG distance or patella alta [9]. Although TTO provides favorable outcomes, the procedure is technically demanding and carries a substantial risk of complications, including nonunion, tibial fracture, fixation failure, infection, neurovascular injury, and symptomatic hardware requiring reoperation [10,11,12]. Implant-related irritation is the most common reason for secondary procedures, with removal rates reported as high as 49% [13,14,15], largely due to the limited soft-tissue coverage over the anterior proximal tibia.

Headless compression screws have been proposed as a low-profile alternative to conventional screws and have been shown in other orthopedic procedures to reduce hardware-related symptoms and the need for removal [16,17]. However, evidence regarding their use in TTO is scarce, with only one study demonstrating a reduction in hardware removal rates [18]. Despite the high rate of hardware-related complications following TTO, there is limited comparative evidence on fixation methods, and the potential benefits of headless screws remain largely unexplored. Given this gap in the literature, the present study aimed to compare hardware removal and complication rates between headless and headed screw fixation in TTO for patellofemoral instability. It was hypothesized that headless screws would reduce the incidence of symptomatic implants and the need for reoperation.

## 2. Materials and Methods

### 2.1. Patients and Study Design

**Figure 1:**
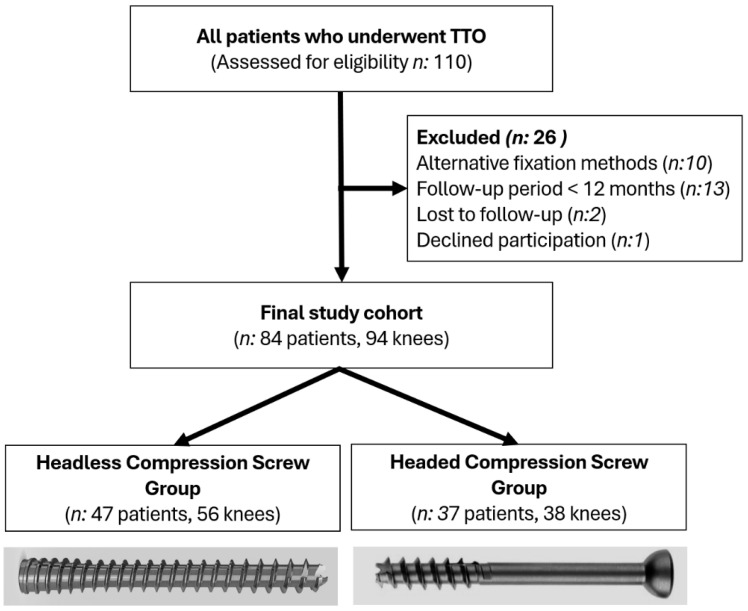
Flow diagram of patient selection.

This retrospective study included patients who underwent TTO for first-time or recurrent patellar dislocation at our institution between 2014 and 2024. A total of 84 patients (94 knees), including 10 who underwent bilateral procedures, met the inclusion criteria of complete clinical records and at least 12 months of radiological follow-up. Patients were excluded if they had incomplete data, a follow-up period shorter than 1 year, were lost to follow-up, declined to participate, or underwent fixation methods other than screw fixation. The patient selection process is illustrated in Figure 1. Ethical approval was obtained from the institutional review board (Date: 26 September 2024, Approval No: 14/12), and all participants provided written informed consent. The study was conducted in accordance with the principles outlined in the Declaration of Helsinki. This study was reported in accordance with the Strengthening the Reporting of Observational Studies in Epidemiology (STROBE) guidelines for observational studies [19].

### 2.2. Surgical Technique and Implants

All procedures were performed with the patient in the supine position under general or spinal anesthesia. A midline longitudinal incision (8–10 cm) was made beginning at the distal patellar tendon insertion. The medial and lateral borders of the patellar tendon were identified, and the tendon was isolated with a clamp passed beneath it. The lateral muscle attachments were elevated subperiosteally and retracted laterally to expose the proximal tibia. An oblique osteotomy of the tibial tubercle was planned, extending 6–8 cm distal to the patellar tendon insertion. After placement of two to three guidewires, the osteotomy was performed at an angle of 30–50° from medial to lateral using an oscillating saw, while preserving the proximal patellar tendon attachment. To protect the tendon, the proximal portion of the osteotomy was completed with an osteotome. The fragment was then transferred medially by approximately 1 cm and fixed with either 4.5 mm partially threaded headed screws, 3.5 mm partially threaded headed screws, or 4.5 mm headless compression screws, according to the surgeon’s preference. No countersinking was performed for headed screws. This was deliberately avoided, especially in the distal part of the osteotomy, where the fragment is thinner, because creating a countersink could further weaken the fragment and increase the risk of fracture. In addition, compression was achieved with a gentle, controlled tightening torque, and overtightening was intentionally avoided to minimize the risk of iatrogenic fracture

Headless screws were introduced during the later phase of the study period as part of evolving institutional practice; therefore, fixation type was not randomized and may be subject to chronological (time-period) bias. When distalization was required, the distal cortical hinge was intentionally disrupted, and the fragment was shortened and repositioned to restore the Caton–Deschamps index. Diagnostic arthroscopy was routinely performed before the osteotomy to evaluate intra-articular structures and treat concomitant pathology. In acute dislocations with osteochondral fragments ≥1 cm, internal fixation was performed; smaller loose bodies were removed arthroscopically. MPFL reconstruction was performed in 86 of 94 knees, and lateral retinacular lengthening was added in 15 cases with excessive patellar tilt. All procedures and intraoperative findings were documented in the operative records. All procedures were performed by a single knee surgeon (corresponding author) using a standardized technique.

### 2.3. Postoperative Follow-Up

Suction drains were removed on the first postoperative day. All patients were immobilized in a hinged long-leg knee brace and allowed partial weight-bearing with two crutches, as tolerated. Straight-leg raises and active range-of-motion (ROM) exercises were initiated in the early postoperative period. Sutures were removed on postoperative day 20. The knee brace was discontinued after six weeks, after which patients progressed to a structured strengthening program. Return to non-contact physical activities, including swimming, cycling, resistance training, and jogging, was permitted once radiographic union was achieved, and knee ROM and quadriceps strength were comparable to the contralateral side. Return to competitive sports was allowed between the sixth and ninth postoperative months, provided patients demonstrated full quadriceps strength, symmetrical ROM, a negative apprehension test, and no subjective instability.

### 2.4. Radiological Assessments and Data Collection

Preoperative imaging included standing anteroposterior (AP) and lateral knee radiographs, full-length weight-bearing lower extremity radiographs, and either magnetic resonance imaging (MRI) or computed tomography (CT) scans, depending on the clinical indication. Trochlear dysplasia was classified according to the Dejour system [20]. Radiographic measurements comprised the TT–TG distance, patellar tilt angle, and patellar height. Patellar height was evaluated using the Caton–Deschamps index, with values > 1.2 defined as patella alta [21]. Mechanical axis deviation (MAD) was determined on full-length lower extremity radiographs: values 0–15 mm medial to the knee center were considered normal, >15 mm medial deviations indicated varus alignment, and lateral deviations indicated valgus alignment. Final follow-up radiographs were assessed in all patients to confirm osteotomy healing. Additional clinical data, including patient age at surgery, sex, laterality, comorbidities, smoking status, American Society of Anesthesiologists (ASA) score, prior knee surgeries, and other demographic characteristics, were retrieved from institutional electronic medical records.

### 2.5. Functional Outcome Assessments

At the final follow-up, all patients were invited to the hospital and underwent a standardized clinical evaluation. This included assessment of knee ROM, manual muscle testing of quadriceps strength relative to the contralateral limb, the apprehension test, evaluation for a J sign, and the patellar grind test. Quadriceps atrophy was quantified by measuring thigh circumference 5 cm proximal to the superior pole of the patella bilaterally. A neurovascular examination was performed in all patients, with particular attention to peri-incisional hypoesthesia. Functional outcomes were assessed using the Kujala Anterior Knee Pain Score [22,23], the Lysholm Knee Scoring Scale [24,25], and the Tegner Activity Score to document pre- and postoperative activity levels. Patient-reported satisfaction was rated on a 10-point Likert scale. Cosmetic evaluation of the surgical scar was performed using a visual analog scale (VAS), ranging from 0 (worst) to 10 (best) [26]. All postoperative clinical examinations at final follow-up were performed by the senior author (O.U) using a standardized assessment protocol; patient-reported outcome questionnaires were completed by patients at the same visit.

### 2.6. Assessment of Complications

All complications observed during the follow-up period were systematically recorded. Because of the retrospective design, radiographic follow-up intervals were not strictly standardized; however, patients were routinely evaluated with anteroposterior and lateral knee radiographs during postoperative visits to monitor the union. Osteotomy union was defined radiographically as bridging trabeculation/cortical continuity across the osteotomy site on both views, with no progressive radiolucent gap, and clinical improvement, such as the absence of focal tenderness at the osteotomy site and the ability to progress to weight bearing. Computed tomography (CT) was obtained when union was equivocal on plain radiographs or when delayed healing was suspected clinically.

Symptomatic (painful) hardware was defined as localized anterior tibial pain and/or focal tenderness on palpation over the screw site, commonly aggravated by kneeling or direct pressure, and attributed clinically to implant prominence/irritation after confirmation of osteotomy union. Implant removal performed for focal screw-site symptoms was labeled as painful hardware removal.

### 2.7. Statistical Analysis

Statistical analyses were performed using SPSS Statistics Base v.23 for Windows. Continuous variables were summarized as the mean ± standard deviation, and categorical variables as frequencies and percentages. The distribution of continuous data was assessed using the Kolmogorov–Smirnov and Shapiro–Wilk tests. Depending on data normality, between-group comparisons were performed with the Student’s t-test or the Mann–Whitney U test, and categorical variables were analyzed using the Chi-square test. Paired preoperative and postoperative values were compared with the Wilcoxon signed-rank test. A *p*-value < 0.05 was considered statistically significant.

## 3. Results

A total of 47 patients (56 knees, including nine bilateral cases) who underwent fixation with headless compression screws and 37 patients (38 knees, including one bilateral case) who underwent fixation with headed compression screws were evaluated. The two groups showed no significant differences in baseline demographic or anatomical characteristics, including age (20.4 ± 6.7 vs. 22.3 ± 10.2 years, *p* = 0.537), sex (*p* = 0.348), BMI (*p* = 0.473), and patellofemoral radiological parameters (Table 1).

The mean duration of surgery was significantly shorter in the headless screw group (95.8 ± 11.6 vs. 106.8 ± 15.9 min, *p* = 0.001). The mean length of hospital stay was also reduced in this group (1.4 ± 0.7 vs. 2.1 ± 0.8 days, *p* = 0.001). Concomitant MPFL reconstruction was performed in 98.2% of knees in the headless screw group compared with 73.7% in the headed screw group (*p* = 0.001). In contrast, lateral retinacular lengthening was more often performed in the headed screw group (36.8% vs. 7.1%, *p* = 0.001). Additional perioperative characteristics are summarized in Table 2.

The mean clinical follow-up was 19.6 ± 5.9 months in the headless screw group and 45.8 ± 24.5 months in the headed screw group (*p* = 0.001). Both groups showed significant improvement in Kujala, Lysholm, and Tegner scores compared with their preoperative values. However, no significant differences were found between groups in postoperative outcomes (Kujala: 94.0 ± 7.6 vs. 92.6 ± 8.7, *p* = 0.650; Lysholm: 94.4 ± 5.6 vs. 94.6 ± 6.2, *p* = 0.782; Tegner: 5.4 ± 1.5 vs. 5.0 ± 1.2, *p* = 0.260). Additional functional outcomes and related clinical findings are presented in Table 3.

**Figure 2:**
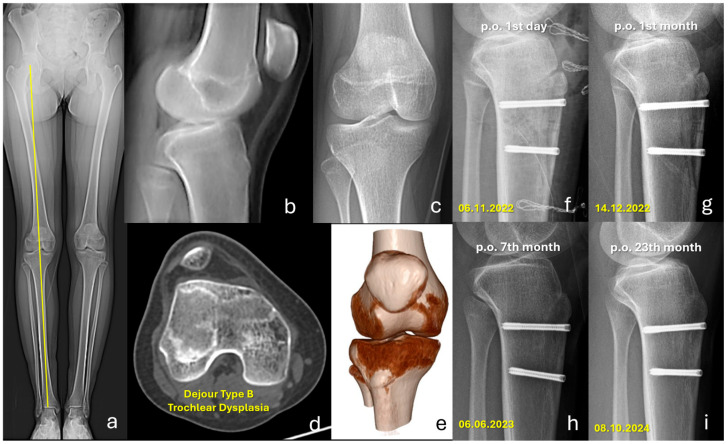
Preoperative imaging and postoperative follow-up of a 13-year-old female who presented with recurrent patellar dislocation. (**a**) Preoperative standing full-length anteroposterior radiograph demonstrating normal lower-limb alignment (yellow line indicates the mechanical axis). (**b**,**c**) Preoperative lateral and anteroposterior knee radiographs. (**d**) Axial computed tomography (CT) image showing a flat trochlea, consistent with Dejour type B trochlear dysplasia. (**e**) Three-dimensional CT reconstruction demonstrating lateralization of the tibial tubercle with an increased tibial tubercle–trochlear groove (TT–TG) distance (22.5 mm). (**f**) Postoperative day 1 radiograph after tibial tubercle osteotomy, fixed with two headless compression screws. (**g**–**i**) Follow-up radiographs at 1, 7, and 23 months postoperatively showing progressive healing and union of the osteotomy site.

Regarding complications, no cases of nonunion or tibial fracture occurred in the headless screw group (Figure 2), whereas one case of each was observed in the headed screw group. The incidence of symptomatic implant removal was significantly lower in the headless group (0% vs. 13.2%, *p* = 0.009), and the overall reoperation rate was also significantly reduced (3.6% vs. 26.3%, *p* = 0.002). Other postoperative complications, including recurrent patellar instability, superficial wound infection, and peri-incisional hypoesthesia, were comparable between groups When isolated symptomatic implant removal was excluded, reoperation rates were 3.6% (n:2) in the headless screw group and 13.2% (n:5) in the headed screw group (*p* = 0.092), indicating that hardware-related reoperations primarily drove the overall between-group difference in reoperation (Table 4).

**Figure 3:**
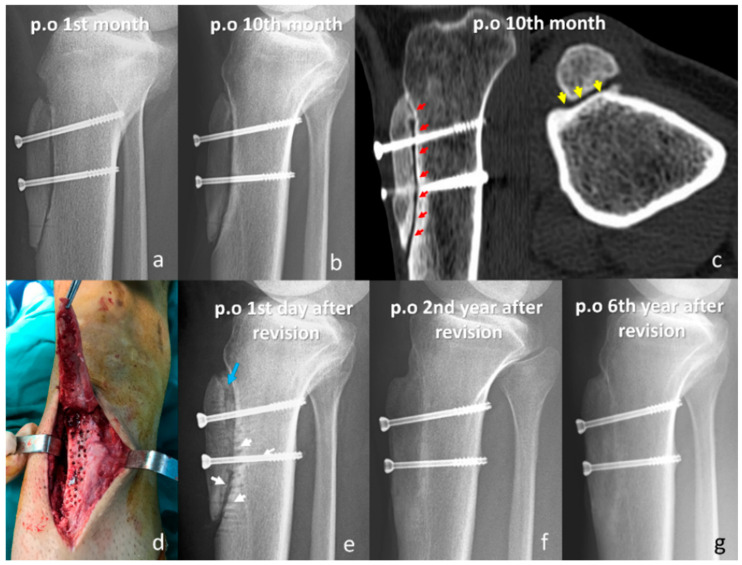
Nonunion case (**a**) Postoperative first-month lateral radiograph showing initial fixation with two-headed screws. (**b**) Tenth-month lateral radiograph indicating a persistent osteotomy line suggestive of nonunion. (**c**) Coronal and axial CT images at 10 months postoperatively demonstrating sclerotic bone edges and radiolucent lines (red arrows) at the osteotomy site, consistent with nonunion. The axial view also shows the established nonunion with a sclerotic osteotomy plane (yellow arrows). (**d**) Intraoperative photograph taken during revision surgery showing fibrous tissue at the osteotomy site and sclerotic bone requiring debridement. (**e**) Immediate postoperative radiograph following revision with autologous iliac crest bone grafting and screw refixation; the blue arrow indicates the autograft, and white arrows show the multiple drilling sites. (**f**) Follow-up radiograph at two years post-revision showing complete union at the osteotomy site. (**g**) Six-year follow-up radiograph demonstrating maintained union and stable fixation without further complications.

**Figure 4:**
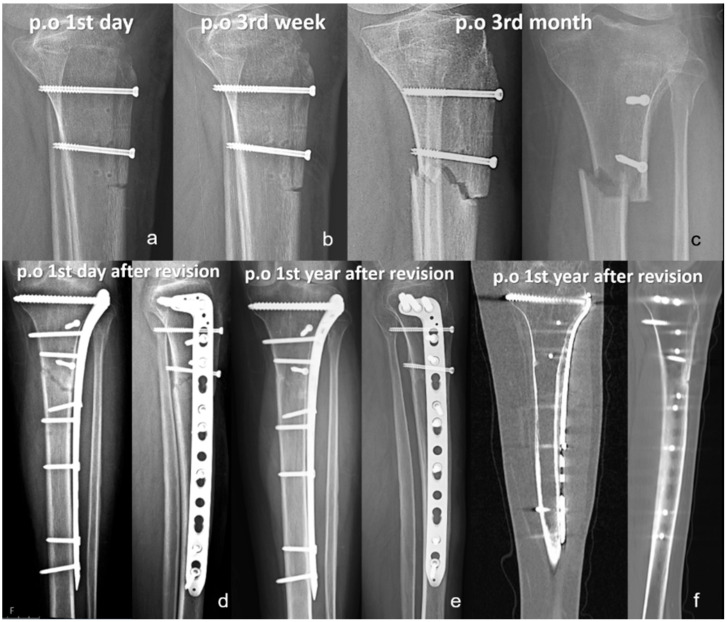
Radiographic follow-up of the patient who developed a tibial fracture after tibial tubercle osteotomy (TTO) and underwent successful revision with plate fixation. (**a**) Postoperative day 1 radiograph showing proper alignment and fixation. (**b**) Third-week radiograph showing maintained stable fixation of the osteotomy. (**c**) Third-month radiograph demonstrating a displaced transverse tibial fracture originating from the distal end of the osteotomy site. (**d**) Immediate postoperative radiographs following open reduction and internal fixation using a proximal tibial locking plate. (**e**) One-year follow-up anteroposterior and lateral radiographs demonstrating complete fracture healing and stable fixation. (**f**) One-year postoperative CT scan confirming cortical continuity and radiological union at both the osteotomy and fracture sites.

In the headless screw group, two reoperations were performed, both for recurrent patellar instability. One patient underwent additional distalization with lateral retinacular lengthening, and the other received isolated lateral lengthening. Stability was restored in both cases, with final Kujala scores of 90 and 98, respectively. In the headed screw group, five reoperations were recorded: one for nonunion, one for tibial fracture, one for arthrofibrosis, and two for recurrent instability. The patient with arthrofibrosis underwent arthroscopic debridement and manipulation under anesthesia, which resulted in complete restoration of the knee range of motion. The tibial fracture was attributed to an imprecise distal osteotomy cut and was sustained after a low-energy fall; it was successfully treated with proximal anatomic tibial plating, resulting in union (Figure 3). In the nonunion case, fibrotic tissue and sclerotic bone were debrided, and an iliac crest autograft was applied prior to revision fixation, resulting in radiographic union at follow-up (Figure 4). Both patients with recurrent instability underwent revision MPFL reconstruction, during which the initially placed TTO screws were removed. Across both groups, seven patients required implant removal; five underwent symptomatic implant removal, whereas two patients had implants removed during revision surgery for another indication. The mean interval between index surgery and hardware removal was 19.8 months (range, 11–35 months). Notably, among patients in whom any 3.5 mm headed screw was used, no symptomatic hardware requiring removal and no osteotomy-related complications were observed; however, formal comparisons by screw diameter were not performed due to mixed configurations and limited sample size.

A post-hoc power analysis based on implant removal rates (0% vs. 18.4%) yielded a statistical power of 98.8% (α = 0.05, two-tailed), indicating that the study was sufficiently powered to detect a significant difference in hardware removal rates between the two groups.

## 4. Discussion

The principal finding of this study was the significantly lower rate of implant removal in patients who underwent TTO with headless screw fixation compared with headed screws. Although both fixation methods yielded similar functional outcomes and overall complication rates, symptomatic hardware requiring removal was not observed in the headless screw group, and the overall reoperation rate was substantially lower. These findings suggest that headless compression screw fixation may offer a preferable option for TTO by reducing hardware-related morbidity and the need for secondary surgery.

A recent retrospective study by Lehane et al. evaluated complications in 476 TTOs performed at a tertiary care center and reported an overall complication rate of 27.5%, with symptomatic hardware requiring removal as the most frequent event (6.5%) [18]. Importantly, hardware removal was significantly more common with headed screws than with headless screws (13.2% vs. 1.7%, *p* < 0.001), consistent with the trend observed in our cohort. However, their analysis did not specifically address headless screw fixation as a primary research question or include a focused comparison of clinical outcomes between screw types. In addition, their patient population was more heterogeneous, incorporating procedures performed for patellofemoral cartilage lesions and pain syndromes, whereas our study analyzed a more uniform cohort of patients with patellofemoral instability. Currently, the study by Lehane et al. remains the only publication to date providing comparative data on the use of headless screws in TTO [18]. Our study builds on these findings by offering a direct, statistically powered comparison of headed versus headless screw fixation, with particular emphasis on implant-related complications and reoperation rates. These results provide new and clinically relevant evidence supporting the potential role of headless screws in reducing symptomatic hardware and the need for implant removal in patellofemoral instability surgery.

Recent studies have highlighted the critical influence of screw head prominence and limited soft-tissue coverage on the development of symptomatic hardware following TTO. In a large retrospective risk analysis, Davis et al. identified low body mass index (BMI) and reduced soft-tissue thickness overlying the screw head as independent predictors of implant removal [27]. Their findings suggest that lean patients, particularly those with minimal subcutaneous cushioning, are more susceptible to hardware irritation from prominent screws. Similarly, Johnson et al. demonstrated that larger-diameter screws (4.5 mm) were associated with significantly higher removal rates than smaller screws (3.5 mm), an effect most pronounced in distalization procedures, where anterior soft-tissue coverage is further diminished [28]. Although neither study specifically evaluated headless screws, both underscore the importance of minimizing anterior tibial prominence to reduce postoperative morbidity. In our series, despite a comparable demographic and anatomical profile, none of the patients treated with headless compression screws required implant removal. This is likely attributable to the ability of headless screws to be inserted flush with or slightly below the cortical surface, thereby reducing the risk of mechanical irritation even in anatomically vulnerable patients. Taken together, these findings provide further support for the use of low-profile fixation, particularly headless screw systems, to mitigate symptomatic hardware in TTO.

From a technical standpoint, the biomechanics of load distribution at the osteotomy surface may differ between fixation constructs. A headed screw, particularly when combined with a washer, can increase the bearing surface and, theoretically, lower local contact pressure, thereby reducing the risk of cut-through or penetration of a thin osteotomy fragment in poor bone quality. In addition, when small bony chips become interposed beneath the head of a headed screw, forcing final tightening with a screwdriver may theoretically create a crack or fracture in the osteotomy fragment; this consideration is one reason why washers may be preferred in selected cases. However, washers also increase hardware prominence and may exacerbate anterior tibial soft-tissue irritation, potentially increasing the risk of symptomatic hardware and subsequent implant removal. For this reason, washers were not used in our series, and headed screws were tightened with a gentle, controlled torque after careful clearance of interposed bony debris. Headless screws, while offering a lower-profile construct that may reduce soft-tissue irritation, could theoretically have a smaller surface area for load distribution at the entry point; nevertheless, we did not observe osteotomy cutout/penetration attributable to headless fixation in this cohort. Future prospective studies comparing standardized constructs (including washer use) would be valuable for better defining these theoretical trade-offs.

An additional consideration is the potential economic cost of fixation constructs. Headless screws are generally more expensive than conventional headed screws; however, a lower rate of symptomatic hardware and subsequent implant removal could offset this difference by avoiding additional surgery and its associated direct and indirect costs. Although our study did not include a formal health-economic evaluation and costs vary across healthcare systems, these findings suggest that the higher upfront implant cost of headless screws may be partially or fully balanced by reduced downstream utilization related to hardware irritation. Future prospective studies incorporating standardized cost inputs and quality-of-life measures are warranted to assess cost-effectiveness more rigorously.

Functional outcomes in this study were comparable between groups, consistent with previous reports indicating that the type of screw fixation does not significantly affect long-term knee function, provided that osteotomy union is achieved and mechanical alignment is restored [13,14]. Importantly, no cases of nonunion or fixation failure were observed in the headless screw group. Biomechanical studies have shown that variable-pitch headless compression screws generate compression forces equivalent to those of conventional headed screws, without compromising axial stability or resistance to failure [29,30]. These findings indicate that reduced thread engagement at the screw head does not imply biomechanical inferiority. Taken together, the results indicate that headless screw fixation offers advantages in minimizing hardware-related morbidity while maintaining mechanical stability and clinical efficacy. Given the consistent association between symptomatic implants and reoperation in TTO, low-profile fixation strategies such as headless compression screws should be considered a valuable option.

The primary strength of this study lies in its direct comparison of two screw fixation techniques for TTO, with clearly defined endpoints related to hardware removal and complication rates. The inclusion of a relatively large cohort and a post hoc power analysis demonstrating adequate statistical power further strengthens the validity of the findings. Nonetheless, several limitations should be acknowledged. First, the retrospective design inherently carries risks of selection and reporting bias. Notably, the fixation method was not randomly allocated; instead, the choice between headed and headless screws reflected routine clinical practice. Therefore, the observed differences, particularly implant-related outcomes, may be confounded by indication and other unmeasured factors, despite broadly comparable baseline characteristics. In addition, between-group differences in concomitant procedures and perioperative variables may have affected outcomes and complicate causal interpretation. The significantly shorter follow-up in the headless screw group may have reduced the likelihood of detecting delayed complications, even though all patients were followed for at least one year. Finally, as a single-center study, the results may be subject to center-specific surgical protocols and may not be generalizable to all settings.

These findings support considering low-profile fixation constructs to reduce symptomatic hardware and subsequent implant removal after tibial tubercle osteotomy, while ensuring stable compression and adherence to meticulous surgical technique (appropriate screw trajectory, avoidance of overtightening, and protection of the distal thin fragment). Patients should be counseled preoperatively that implant-related symptoms may necessitate secondary surgery, particularly with prominent-headed constructs. For researchers, future work should focus on prospective comparative studies with standardized indications and concomitant procedures, predefined radiographic follow-up intervals, and longer follow-up to capture late complications. Incorporating patient-centered outcomes and health-economic analyses will be essential to determine whether higher implant acquisition costs are offset by reduced downstream utilization related to hardware removal.

## 5. Conclusions

In conclusion, headless screw fixation in tibial tubercle osteotomy was associated with significantly lower rates of painful implant removal and overall reoperation compared to conventional headed screws, without compromising functional outcomes. These findings support the use of headless compression screws as a reliable and potentially superior method of fixation in the surgical treatment of patellofemoral instability. Further prospective studies with long-term follow-up and randomized designs are warranted to validate these results and guide clinical decision-making.

## Figures and Tables

**Table 1:** Comparison of demographic and clinical characteristics of patients. *Abbreviations: SD: Standard deviation, BMI: Body mass index, ASA: American Society of Anesthesiologists. #: Number*.

Variables	Headless Screws (# of Knees: 56)	Headed Screws (# of Knees: 38)	*p*-Value
Age (years ± SD)	20.4 ± 6.7	22.3 ± 10.2	0.537 ^1^
Sex (n, %)			0.348 ^2^
*Male*	13 (23.2%)	11 (28.9%)	
*Female*	43 (76.8%)	27 (71.1%)	
Side			0.480 ^2^
*Right*	25 (44.6%)	18 (47.4%)	
*Left*	31 (55.4%)	20 (52.6%)	
Weight (kg ± SD)	65.2 ± 12.6	66.4 ± 12.7	0.656 ^1^
Height (cm ± SD)	165.2 ± 8.6	164.6 ± 9.6	0.648 ^3^
BMI (kg/m^2^ ± SD)	23.9 ± 4.4	24.6 ± 5.1	0.473 ^3^
Acute vs. Recurrent (n, %)			0.331 ^2^
*Acute*	17 (30.4%)	14 (36.8%)	
*Recurrent*	39 (69.6%)	24 (63.2%)	
Tobacco users (n, %)	10 (17.9%)	8 (21.1%)	0.448 ^2^
Diabetes Mellitus (n, %)	1 (1.8%)	0 (0%)	0.596 ^2^
ASA Score			0.662 ^2^
*ASA I*	41 (73.2%)	27 (71.1%)	
*ASA II*	14 (25.0%)	11 (28.9%)	
*ASA III*	1 (1.8%)	0 (0%)	
TT-TG Distance (mm ± SD)	20.6 ± 3.5	19.9 ± 3.9	0.355 ^3^
Dejour Classification (n, %)			0.103 ^3^
*Type A*	15 (26.8%)	5 (13.2%)	
*Type B*	17 (30.4%)	15 (39.5%)	
*Type C*	15 (26.8%)	6 (15.8%)	
*Type D*	9 (16.1%)	12 (31.6%)	
Caton–Deschamps Index	1.16 ± 0.17	1.11 ± 0.16	0.190 ^3^
Patella Alta (n, %)	21 (37.5%)	12 (31.6%)	0.357 ^2^
Patellar Tilt (° ± SD)	31.3 ± 10.9	31.7 ± 10.6	0.860 ^3^
Limb Alignment (n, %)			0.351 ^2^
*Normal*	35 (62.55)	21 (55.3%)	
*Varus*	9 (16.1%)	4 (10.5%)	
*Valgus*	12 (21.4%)	13 (34.2%)	
Prior Surgery (n, %)	4 (7.1%)	4 (10.5%)	0.414 ^2^

^1^ Mann–Whitney U test, ^2^ Chi-Square Test, ^3^ Student’s T-test.

**Table 2:** Comparison of perioperative characteristics of patients. *Abbreviations: MPFL: Medial patellofemoral ligament, MPFLR: Medial patellofemoral ligament reconstruction, OCF: Osteochondral fragment, AHPLT: Anterior half of peroneus longus tendon, LOS: Length of hospital stay. #: Number*.

Variables	Headless Screws (# of Knees: 56)	Headed Screws (# of Knees: 38)	*p*-Value
Length of Osteotomy (cm ± SD)	7.9 ± 0.8	7.5 ± 1.5	0.067 ^1^
Osteotomy Shape (n, %)			0.001 ^2^
*Greenstick*	43 (76.8%)	17 (44.7%)	
*Slopped*	13 (23.2%)	4 (10.5%)	
*Blunt*	0 (0%)	17 (44.7%)	
Number of Screws (n, %)			0.001 ^2^
*Two-screw fixation*	20 (35.7%)	31 (81.6%)	
*Three-screw fixation*	36 (64.3%)	7 (18.4%)	
Screw Size ** (n, %)			0.011 ^2^
*3.5 mm*	-	7 (8.5%)	
*4.5 mm*	148 (100%)	76 (91.5%)	
Concomitant interventions (n, %)			
*MPFLR*	55 (98.2%)	31 (73.7%)	0.007 ^2^
*Lateral Lengthening*	5 (8.9%)	14 (36.8%)	0.001 ^2^
*Patellar OCF Removal*	3 (5.4%)	2 (5.3%)	0.679 ^2^
*Patellar OCF Fixation*	1 (1.8%)	2 (5.3%)	0.357 ^2^
*Distalization*	1 (1.8%)	3 (7.9%)	0.300 ^2^
MPFL Graft Option * (n, %)			0.564 ^2^
*Gracilis*	54 (98.2%)	27 (96.4%)	
*AHPLT*	1 (1.8%)	1 (3.6%)	
Double vs. Single Bundle MPFLR *			0.548 ^2^
*Single*	48 (87.3%)	25 (89.3%)	
*Double*	7 (12.7%)	3 (10.7%)	
Duration of Operation (min ± SD)	95.8 ± 11.6	106.8 ± 15.9	0.001 ^1^
Anesthesia type			0.520 ^2^
*Spinal*	49 (87.5%)	34 (89.5%)	
*General*	7 (12.5%)	4 (10.5%)	
LOS (days ± SD)	1.4 ± 0.7	2.1 ± 0.8	0.001 ^1^

^1^ Mann–Whitney U test, ^2^ Chi-square test * Only patients who underwent MPLR were compared. ** Number of screws were compared.

**Table 3:** Comparison of functional outcomes of the patients. *Abbreviations: SD: Standard deviation, ROM: Range of motion, NA: Not applicable. #: Numbe*.

Variables	Headless Screws (# of Knees: 56)	Headed Screws (# of Knees: 38)	*p*-Value
Clinical Follow-up (months ± SD)	21.8 ± 6.7	62.1 ± 30.3	0.001 ^1^
Radiological Follow-up (months ± SD)	19.6 ± 5.9	45.8 ± 24.5	0.001 ^1^
Preop Kujala Score (points ± SD)	55.4 ± 19.7	49.3 ± 20.0	0.176 ^1^
Postop Kujala Score (points ± SD)	94.0 ± 7.6	92.6 ± 8.7	0.650 ^1^
*p*-value *	0.001 ^2^	0.001 ^2^	
Preop Lysholm Knee Score (points ± SD)	58.2 ± 15.8	53.8 ± 16.9	0.228 ^1^
Postop Lysholm Knee Score (points ± SD)	94.4 ± 5.6	94.6 ± 6.2	0.782 ^1^
*p*-value *	0.001 ^2^	0.001 ^2^	
Preop Tegner Activity Scale (points ± SD)	5.9 ± 1.8	6.1 ± 1.7	0.697 ^1^
Postop Tegner Activity Scale (points ± SD)	5.4 ± 1.5	5.0 ± 1.2	0.260 ^1^
*p*-value *	0.016 ^2^	0.001 ^2^	
Postop Tegner Activity Scale			0.454 ^3^
*Remained unchanged*	35 (62.5%)	19 (50.0%)	
*Decreased*	18 (32.1%)	17 (44.7%)	
*Increased*	3 (5.4%)	2 (5.3%)	
Knee ROM Deficit (n, %)			NA
*Flexion*	0 (0%)	0 (0%)	
*Extension*	0 (0%)	0 (0%)	
Manual Muscle Strength Deficit (n, %)	0 (0%)	0 (0%)	NA
Thigh Atrophy	0 (0%)	0 (0%)	NA
J-Sign at Final Follow-up (n, %)			0.449
*None*	45 (80.4%)	33 (86.8%)	
*Mild*	9 (16.1%)	5 (13.2%)	
*Moderate*	2 (3.6%)	0 (0%)	
*Severe*	0 (0%)	0 (0%)	
Positive Apprehension Test (n, %)			0.212 ^3^
*Mild*	6 (10.7%)	3 (7.9%)	
*Moderate*	2 (3.6%)	5 (13.2%)	
Positive Giving way (n, %)	0 (0%)	1 (2.6%)	0.404 ^3^
Positive Patellar Grinding Test (n, %)	0 (0%)	0 (0%)	NA
Overall Satisfaction (points ± SD)	9.2 ± 1.1	9.0 ± 2.3	0.262 ^1^
Cosmetic Satisfaction (points ± SD)	7.6 ± 2.3	7.5 ± 2.5	0.925 ^1^

^1^ Mann–Whitney U test, ^2^ Related Samples Wilcoxon Signed Rank test, ^3^ Chi-Square Test, * *p*-values on the rows are comparisons within the same group, between preoperative and final follow-up values.

**Table 4:** Comparison of complications and revision between groups. *Abbreviations, NA: Not applicable. #: Numbe*.

Variables	Headless Screws (# of Knees: 56)	Headed Screws (# of Knees: 38)	*p*-Value
Non-Union (n, %)	0 (0%)	1 (2.6%)	0.404 ^1^
Fracture (n, %)	0 (0%)	1 (2.6%)	0.404 ^1^
Recurrent Instability (n, %)	2 (3.6%)	2 (5.3%)	0.534 ^1^
Superficial Infection (n, %)	2 (3.6%)	1 (2.6%)	0.799 ^1^
Deep Infection (n, %)	0 (0%)	0 (0%)	NA
Pulmonary Embolism (n, %)	0 (0%)	1 (2.6%)	0.404 ^1^
Hypoesthesia (n, %)	27 (48.2%)	13 (34.2%)	0.127 ^1^
Arthrofibrosis (n, %)	0 (0%)	1 (2.6%)	0.404 ^1^
Hypertrophic Scar/Keloid (n, %)	0 (0%)	0 (0%)	NA
Painful Implant Removal (n, %)	0 (0%)	5 (13.2%)	0.009 ^1^
Reoperation excluding implant removal (n, %)	2 (3.6%)	5 (13.2%)	0.092 ^1^
Reoperation for any reason (n, %)	2 (3.6%)	10 (26.3%)	0.002 ^1^

^1^ Chi-square test.

## Data Availability

The datasets are not publicly available. The de-identified data are available upon request to the corresponding author due to privacy, ethical, and legal restrictions protecting patient confidentiality.

## Abbreviations

The following abbreviations are used in this manuscript:

TTO	Tibial Tubercle Osteotomy
PFI	Patellofemoral Instability
MPFL	Medial Patellofemoral Ligament
MPFLR	Medial Patellofemoral Ligament Reconstruction
TT-TG	Tibial Tuberosity-Trochlear Groove
ROM	Range of Motion
MRI	Magnetic Resonance Imaging
CT	Computed Tomography
MAD	Mechanical Axis Deviation
ASA	American Society of Anesthesiologists
VAS	Visual Analog Scale
